# Ceramic composite with gentamicin decreases persistent infection and increases bone formation in a rat model of debrided osteomyelitis

**DOI:** 10.5194/jbji-6-283-2021

**Published:** 2021-07-20

**Authors:** Aleksey Dvorzhinskiy, Giorgio Perino, Robert Chojnowski, Marjolein C. H. van der Meulen, Mathias P. G. Bostrom, Xu Yang

**Affiliations:** 1 Department of Orthopaedic Surgery, Hospital for Special Surgery, New York, NY 10021, USA; 2 Department of Biomedical Engineering, Cornell University, Ithaca, NY 14853, USA

## Abstract

**Introduction**: Current methods of managing osteomyelitic voids after debridement are inadequate and result in significant morbidity to patients. Synthetic ceramic void fillers are appropriate for non-infected bone defects but serve as a nidus of re-infection in osteomyelitis after debridement. CERAMENT G (CG) is an injectable ceramic bone void filler which contains gentamicin and is currently being evaluated for use in osteomyelitic environments after debridement due to its theoretical ability to serve as a scaffold for healing while eliminating residual bacteria after debridement through the elution of antibiotics. The goal of this study was to evaluate (1) the rate of persistent infection and (2) new bone growth of a debrided osteomyelitic defect in a rat model which has been treated with either gentamicin-impregnated ceramic cement (CERAMENT G) or the same void filler without antibiotics (CERAMENT, CBVF). **Methods**: Osteomyelitis was generated in the proximal tibia of Sprague Dawley rats, subsequently debrided, and the defect filled with either (1) CG (n=20), (2) CBVF (n=20), or (3) nothing (n=20). Each group was euthanized after 6 weeks. Infection was detected through bacterial culture and histology. Bone growth was quantified using microCT. **Results**: Infection was not detected in defects treated with CG as compared with 35 % of defects (7/20) treated with CBVF and 50 % (10/20) of empty defects (p=0.001). Bone volume in the defect of CG-treated rats was greater than the CBVF (0.21 vs. 0.17, p=0.021) and empty groups (0.21 vs. 0.11, p<0.001) at 6 weeks after implantation. **Conclusions**: Ceramic void filler with gentamicin (CERAMENT G) decreased the rate of persistent infection and increased new bone growth as compared to the same void filler without antibiotics (CERAMENT) and an empty defect in a rat model of debrided osteomyelitis.

## 1 Introduction

Osteomyelitis is an infectious disease of bone which is often caused by methicillin-sensitive *Staphylococcus aureus* (Aragón-Sánchez et al., 2009; Hatzenbuehler and Pulling, 2011; Lew and Waldvogel, 1997, 2004). While acute osteomyelitis is generally responsive to intravenous antibiotics, chronic osteomyelitis requires surgical debridement due to the presence of necrotic bone (Eckardt et al., 1994; Norden et al., 1992; Stengel et al., 2001). Large segments of dead bone, termed sequestra, can become avascular and harbor invasive organisms thus preventing adequate treatment with intravenous antibiotics. In addition, *S. aureus* invades the osteocyte lacuno-canalicular network (OLCN) of live cortical bone during chronic osteomyelitis (de Mesy Bentley et al., 2017, 2018). Moreover, these bacteria are not susceptible to local high-dose antibiotic treatment in mouse models of osteomyelitis (Masters et al., 2020; Schwarz et al., 2021). Proper debridement requires removal of all necrotic tissue, which can leave a large defect that must be managed to both prevent the recurrence of infection and provide mechanical stability (Davis, 2005; Eckardt et al., 1994; Lew and Waldvogel, 2004; Parsons and Strauss, 2004).

Many approaches to dead space management exist: healing by secondary intention, closed irrigation, temporary antibiotic-laden polymethylmethacrylate (PMMA) beads, and autologous bone grafts (Parsons and Strauss, 2004). None of these options are ideal. Healing by secondary intention and closed irrigation systems have high rates of recurrent infection (Cierny, 1990; Clawson et al., 1973; Kelly et al., 1970). Antibiotic-laden polymethylmethacrylate beads are effective at eradicating infections but require a second procedure for removal and subsequent management of the ensuing defect (Adams et al., 1992; Cierny, 1990). Autologous and allogeneic bone grafts are the most commonly used void fillers, but limited bone stock, concern for disease transmission, and donor site morbidity preclude their use in many situations (Lew and Waldvogel, 2004). Other solutions such as vascularized bone flaps and bone transport mechanisms are cumbersome, expensive, and more suited for larger, segmental defects (Green, 1991; Ilizarov, 1989; Weiland et al., 1984; Wood et al., 1985).

A synthetic alternative that retains the osteoconductive and mechanical qualities of autologous bone graft while also being abundant may be of use in the management of osteomyelitic voids. Ceramic cements composed of hydroxyapatite and calcium sulfate (e.g., CERAMENT Bone Void Filler, CBVF) have been developed which could be superior to PMMA because they are resorbable, osteoconductive, and biologic, thus obviating the need for subsequent removal (Nilsson et al., 2004). Unfortunately, use of these composites in infected environments is not advisable because of their propensity to serve as a nidus of continued infection. Similarly, other ceramic composites consisting of calcium phosphate (Norian SRS), β-tricalcium phosphate (Vitoss^®^), coralline hydroxyapatite and calcium carbonate (Pro Osteon^®^), and calcium sulfate (BonePlast^®^ and OSTEOSET^®^) exist and have their own unique properties, but all are contra-indicated in infected environments (Ricciardi and Bostrom, 2013). Bacterial colonization of these materials could be overcome by impregnating the cement with antibiotics such as gentamicin, a cheap, broad spectrum compound with a low rate of microbial resistance (Klemm, 2001). A new formulation of a calcium sulfate-hydroxyapatite cement impregnated with gentamicin (CERAMENT G, CG) has been developed but is not yet approved by the United States Food and Drug Administration (FDA) for human use. The advantages of using CERAMENT as a drug delivery device are that it forms a paste that can be injected into bone defects, completely filling the cavity and excluding any dead space, which obliterates any areas that may harbor residual bacteria or small fragments of biofilm (Ferguson et al., 2017; McNally et al., 2016). Other commercially available ceramic formulations with impregnated antibiotics include Herafill-G^®^ (calcium sulfate and calcium carbonate) and OSTEOSET^®^ (calcium sulfate with tobramycin) (Oliver et al., 2020) but are non-injectable and therefore may be less ideal for eradication of infection. In addition, the biodegradability of the product allows single-stage surgery and has previously been demonstrated to be highly efficient for treatment of chronic bone infections when combined with debridement and systemic antimicrobials in clinical studies (McNally et al., 2016; Niazi et al., 2019; Oliver et al., 2020). While CERAMENT G has shown overall effectiveness in humans, our study represents the first evaluation of CERAMENT G in a small animal model of debrided osteomyelitis and thus is useful in quantifying the ability of this compound to eradicate infection as well as facilitate new bone growth.

The aim of this study was to evaluate the hypotheses that the ceramic cement with gentamicin (CG) would (1) decrease the rate of persistent infection and (2) increase new bone growth in a rat model of debrided osteomyelitis as compared to the same cement without gentamicin (CERAMENT, CBVF) or an empty mechanically stable defect.

## 2 Materials and methods

### 2.1 Preparation of inoculum

A week prior to the first surgical date, a strain of *Staphylococcus aureus* known to be methicillin- and gentamicin-sensitive (ATCC 29213, American Type Culture Collection, Manassas, VA) was revived from a -80 ${}^{\circ }$C freezer and incubated at 37 ${}^{\circ }$C for 24 h on a tryptone soy agar plate. On the day before surgery, a single colony from a *S. aureus* streak plate was selected and inoculated into tryptic soy broth. This culture was then incubated for 16 h at 37 ${}^{\circ }$C with shaking agitation. On the morning of the induction of osteomyelitis, a subculture of *S. aureus* was made and grown for several hours until reaching an absorbance of 0.6 at 600 nm, which correlates to a viable count of approximately $1.5\times 10^{8}$ bacteria. This was then kept on ice until needed during surgery.

**Figure 1:**
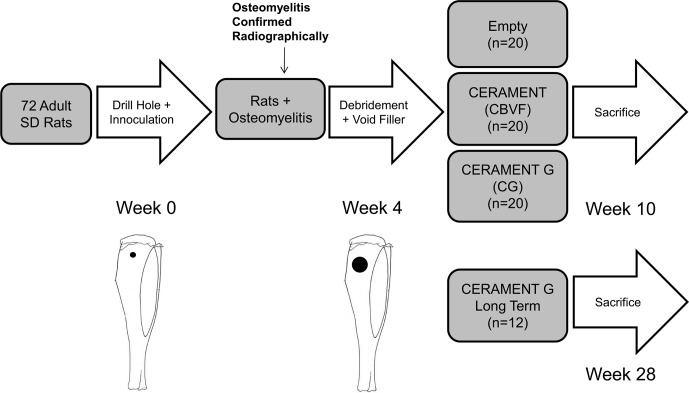
Study design: *S. aureus* ($1.5\times 10^{6}$ CFU) was injected into a drill hole in the right tibia of adult Sprague Dawley rats (n=72). After 3 weeks, the osteomyelitic defect was debrided and treated with (1) CERAMENT G (n=32), (2) CERAMENT (n=20), or (3) nothing (n=20); 6 weeks after the second surgery, 20 rats from each group were euthanized, and the right tibias were harvested. To determine the long-term outcome of the treatment, another group (n=12) of CERAMENT G-treated rats was also euthanized at 6 months after the debridement and treatment.

### 2.2 Induction of osteomyelitis

Using a protocol approved by the Institutional Animal Care and Use Committee of our institution, we carried out this study using a previously described and published rat model of a mechanically stable osteomyelitic defect (Fukushima et al., 2005; Zelken et al., 2007). Our overall study design is shown in Fig. 1. We chose to utilize a model without the use of a sclerosing agent or a foreign body in order to induce a local mechanically stable, consistently sized osteomyelitis in the proximal tibia for ease of debridement and standardized evaluation of the region of interest (Fukushima et al., 2005). Adult male Sprague Dawley rats weighing ∼350 g (n=81, Harlan Laboratories, Indianapolis, Indiana) were anesthetized using isoflurane through inhalational anaesthesia in the animal operating room. Subsequently, under sterile conditions, animals received a drill hole (1 mm diameter, 3 mm depth) in the proximal tibia which was inoculated with $1.5\times 10^{6}$ CFU (10 µL) of gentamicin-sensitive *S. aureus*. The center of the defect was positioned 3 mm inferior to the growth plate and 3 mm lateral to the lateral edge of the tibial tuberosity. The depth of the drill hole was controlled with a 3 mm stop on the end of the drill. Prior to randomization, during the surgery to induce osteomyelitis, nine animals perished due to an error in anaesthesia protocol. No further animals perished prior to the completion of the experiment. Thus, the final sample size was n=72. Animals were housed with a single other companion in clear plastic cages containing bedding as well as free access to water and food. Subcutaneous buprenorphine was administered during the perioperative period for analgesia. Animals were assessed clinically and weighed three times per week. All animals maintained weight throughout the study period and did not display signs of clinical infection.

### 2.3 Confirmation of infection

After 3 weeks, the animals were examined, and the right hind legs were imaged with high-resolution X-ray (Faxitron X-ray Corp, Wheeling, IL). Osteomyelitis was confirmed using an established scoring system which evaluated the presence of osteomyelitis by detection of sequestrum, involucrum, osteolysis, soft tissue swelling and joint effusion (Norden et al., 1980; Rissing et al., 1985; Zelken et al., 2007). Animals positive for at least three of these findings were considered to have developed osteomyelitis. All animals used in this study (n=72) were positive for osteomyelitis in the operative limb prior to randomization.

### 2.4 Description of tested materials

**Figure 2:**
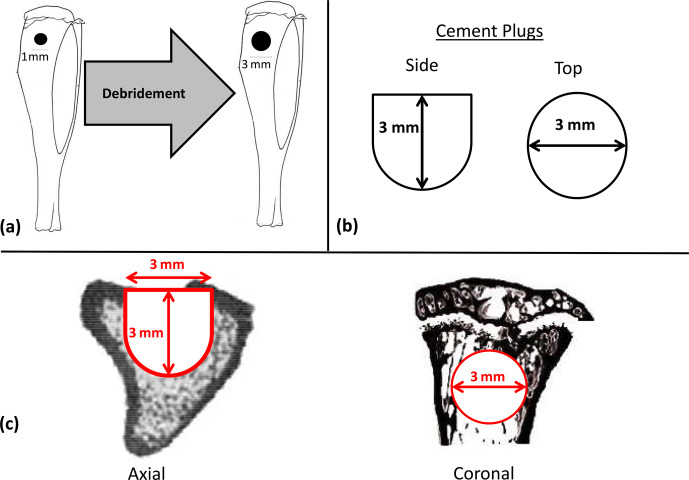
Diagrams of experimental materials and regions of interest. After confirmation of osteomyelitis, all animals underwent debridement using a 3 mm diameter drill centered on the previous 1 mm diameter hole used to create the osteomyelitic cavity with a depth stop of 3 mm **(a)**. Afterwards animals randomized to receive CBVF or CG were filled with their respective plugs **(b)**. During microCT testing, the region of interest (ROI) mimicked the contours of the original implant in its dimensions, which were 3 mm in diameter to a depth of 1.8 mm, at which point the remaining 1.2 mm of depth was tapered to a diameter of 1.5 mm **(c)**.

Both CERAMENT (CBVF) and CERAMENT G (CG) were provided by the sponsoring company (BONESUPPORT AB, Lund, Sweden) in sealed, sterile packaging as prefabricated plugs (3 mm in diameter, 2.98 mm in length). CERAMENT plugs consisted of 40 % hydroxyl apatite and 60 % calcium sulfate. CERAMENT G plugs had identical compositions to CBVF plugs with the exception of 0.29 mg of gentamicin added in the mixture (17.5 mg/mL of paste). This is identical to the composition of both commercially available compounds (Fig. 2b).

### 2.5 Debridement and implantation

After confirmation of osteomyelitis, all animals underwent debridement and implantation. Debridement of the defect was performed using a 3 mm diameter drill, with a depth stop at 3 mm (Fig. 2a). Copious amounts of normal saline were used for irrigation. After debridement and re-draping and disinfecting all instruments, animals were randomized using block randomization to have the defect filled with (1) CERAMENT G, CG (n=32), (2) CERAMENT, CBVF (n=20), or (3) empty (no filler, n=20) (Fig. 1). After randomization, individuals analyzing subsequent data were blinded to experimental group until the completion of the study. Skin incisions were closed with absorbable suture. Once again, subcutaneous buprenorphine was administered during the perioperative period for analgesia. Animals were assessed clinically and weighed three times per week. All animals maintained weight throughout the study period and did not display signs of clinical infection.

No systemic antibiotics were administered either orally or intravenously. Six weeks after the implantation surgery, 20 rats from each group were euthanized using carbon dioxide asphyxiation. Six months after the implantation surgery, the remainder of the CG group rats (n=12) were euthanized to serve as the long-term CG group.

### 2.6 Microbiology

All procedures for tissue collection were carried out using standard sterile technique. The right tibias and surrounding muscle of all animals were harvested immediately after sacrifice. The drill hole was exposed, and the tibia was cut transversely 5 mm distal to the infection site. This was done in such a way to expose both the original inoculation site and the intramedullary canal to sonication and thus provided a microbiological culture of both sites. The samples were vortexed and sonicated in phosphate-buffered saline (PBS) to dislodge the bacteria from the bone and into the PBS solution. The tibias were then removed from the phosphate-buffered saline and placed in 10 % formalin for subsequent microCT and histological analysis. The PBS sonicate fluid was serially diluted and afterwards plated on 5 % sheep blood tryptic soy agar (Sigma-Aldrich, St. Louis, MO). The specimens were then incubated for 48 h and underwent colony enumeration as per standard microbiological protocol (Breed and Dotterrer, 1916). The limit of detection was set at 150 CFU per milliliter. Six random contralateral limbs (CG: 1, CBVF: 2, empty: 3) were also selected as negative controls and tested in this manner for quality control of our microbiological procedure.

### 2.7 Microcomputed tomography (microCT)

The tibias in formalin were kept at room temperature for 1 d, placed at 4 ${}^{\circ }$C for 2 d and then switched to 70 % alcohol. The samples were scanned by microCT (μCT 35; SCANCO Medical, Bassersdorf, Switzerland). The region of interest (ROI) mimicked the contours of the original implant in its dimensions, which were 3 mm in diameter to a depth of 1.8 mm, at which point the remaining 1.2 mm of depth was tapered to a diameter of 1.5 mm. A global threshold was used to exclude any unmineralized tissue or cement. The bone volume fraction (BVF), defined as the bone volume divided by the total volume of the drill hole (BV / TV), was analyzed and compared among groups (Fig. 2c).

### 2.8 Histology

All samples underwent decalcification with 10 % EDTA in phosphate-buffered saline and were embedded in paraffin through serial dehydration. Axial, 7 µm sections were taken at the level of the center of the drill hole and went on to hematoxylin and eosin (H&E) and immunohistochemical staining.

H&E staining was utilized to detect infection in the ROI. The presence of infection in each sample was assessed by recording the presence or absence of histological evidence of osteomyelitis in the form of a neutrophil-predominant response in addition to necrosis of the bone as judged by a single blinded attending musculoskeletal pathologist (Giorgio Perino). This clinical method detects osteomyelitis with a sensitivity of 43 %–84 % and a specificity of 93 %–97 % (Abdul-Karim et al., 1998; Lew and Waldvogel, 2004). Immunohistochemistry was performed using anti-pro-collagen I antibodies (SP1.D8, Developmental Studies Hybridoma Bank, Iowa City, IA) as a marker of osteoblasts and anti-cathepsin K antibodies as a marker of osteoclasts (Zenger et al., 2007). Images of sections were obtained at 100× magnification using a slide scanner (Zeiss EVO 50 SEM, Carl Zeiss Microscopy, LLC, Thornwood, NY). The total numbers of pro-collagen I-positive and cathepsin K-positive cells were manually counted within the area of the previous defect, normalized by bone area, and the results were expressed as osteoblast or osteoclast numbers per square millimeter.

### 2.9 Statistical analysis

Differences in microCT, as well as immunohistochemical counts between treatments, were assessed with one-way ANOVA followed by a Student–Newman–Keuls post hoc method. Proportions of infected samples were compared using chi-squared analysis (histology) and Fisher's exact test (culture) on a 2×4 contingency table. Descriptive statistics were gathered for the long-term group in terms of histological or microbiological evidence of osteomyelitis but were not compared to the others as it did not have a control group at the same time point. The level of significance was p<0.05.

Power was calculated using previous studies that estimated a mean healing response (bone growth) of 6.4 % for the control group and 14.2 % for the CBVF-filled defects with a coefficient of variation of 60 % based on internal data provided by the company. The sample size required to detect such a difference using Satterthwaite's t test with 5 % significance level and 80 % statistical power was 16 rats in each group. The sample size was increased to 20 per group as the assumptions about the average percent bone growth in CG-filled defects compared with CBVF were uncertain.

**Figure 3:**
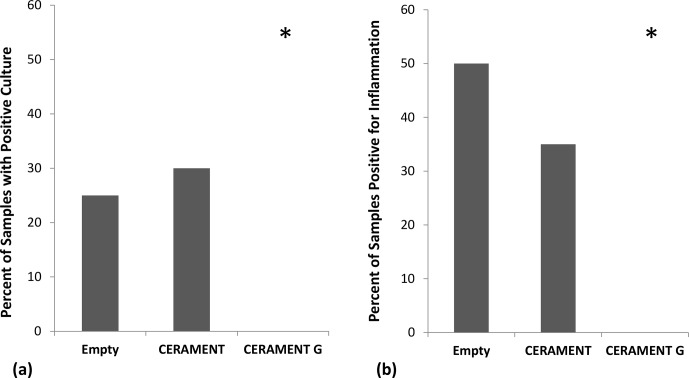
Detection of persistent infection: percentage of samples in each group positive for growth (n=20 per group). CERAMENT G inhibited bacterial growth in the osteomyelitic defect as seen by the percentage of samples in each group that had positive growth on culture medium **(a)** and positive histological evidence of osteomyelitis **(b)**. *p<0.05 using 2×3 contingency table and Fisher's exact test.

**Figure 4:**
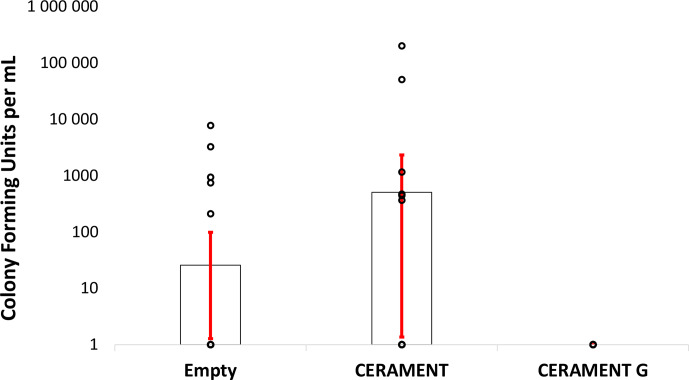
Bacterial counts: colony forming units per milliliter of sonicate taken from empty, CERAMENT Bone Void Filler, and CERAMENT G groups (n=20 per group). Bacterial quantities were not significantly different between treatment groups owing to the large standard deviations of the non-CERAMENT G groups. Error bars represent ±1 standard deviation.

## 3 Results

### 3.1 Bacterial culture (Figs. 3a and 4)

Bacterial cultures were positive in 25 % (5/20) of animals left with an empty defect, 30 % (6/20) of animals treated with CBVF, and 0 % (0/20) of the animals treated with CG (Fig. 3a). The CG group was found to have significantly fewer positive cultures than either the CBVF or empty control groups (p=0.002). The proportion of positive cultures in the CBVF group was not significantly different than the empty group. None of cultures taken from contralateral limbs (negative controls) were found to be positive. Bacterial colony counts trended towards being higher in CBVF-treated animals than in animals left with an empty defect but did not reach significance due to a large standard deviation in colony forming units per milliliter (CFU per milliliter) in the non-CG groups (Fig. 4). None of the long-term group animals filled with CG had positive cultures at 6 months after implantation.

### 3.2 Histology (Figs. 3b and 5)

Histological evidence of osteomyelitis (i.e., a neutrophil-predominant inflammatory reaction ± bone necrosis) was found in 50 % (10/20) of animals with empty defects, 35 % (7/20) of animals treated with CBVF, and none (0/20) of the animals treated with CG (Fig. 3b). The proportion of animals with histological evidence of osteomyelitis in the CG group was significantly lower than animals in both the CBVF and empty groups (p=0.001). The proportion of animals with histological evidence of osteomyelitis in the CBVF group was not different from the empty control group. No histological evidence of osteomyelitis was found in any animals in the CG-treated long-term group. The proportion of animals with positive cultures was not significantly different than the proportion of samples with histological evidence of osteomyelitis within the respective groups (p=0.20). Osteoblast and osteoclast counts were not different between the short-term treatment groups (empty, CBVF or CG).

**Figure 5:**
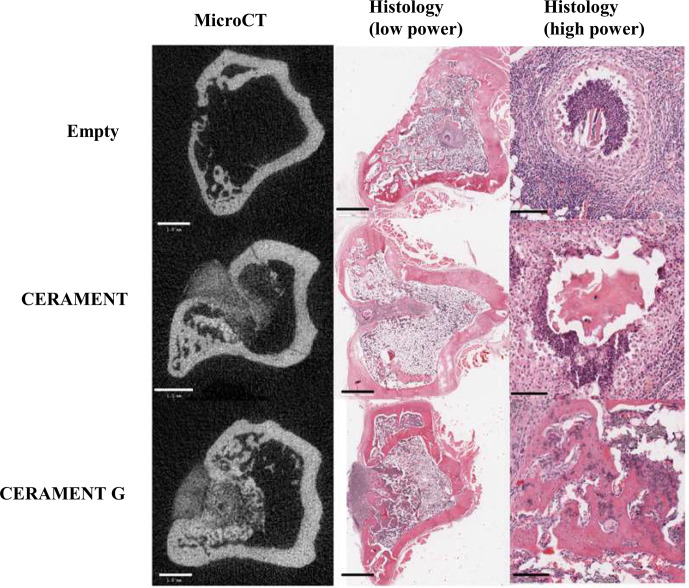
Representative microCT and low/high-magnification H&E sections of representative samples in empty, CERAMENT, and CERAMENT G groups. MicroCT and low-power images show significant bone growth in the previous defect in CERAMENT and CERAMENT G groups. High-power histology images show evidence of persistent osteomyelitis in the empty and CERAMENT groups as evidenced by a neutrophil predominant infiltrate and necrotic bone with empty lacunae. No such reaction is seen in the CERAMENT G group. Scale bars on the bottom left show 1 mm in microCT and low-magnification histology. Scale bars in high-magnification histology show 100 µm.

**Figure 6:**
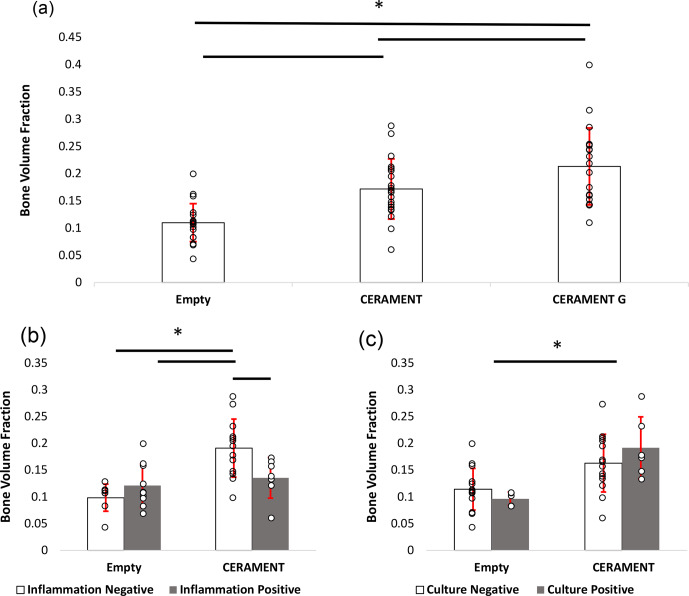
Measurement of new bone growth in empty, CERAMENT, and CERAMENT G groups (n=20): CERAMENT G increased new bone growth compared to empty and CERAMENT groups **(a)**. Animals in the CERAMENT group with histologically evident osteomyelitis formed significantly less bone than those that did not have osteomyelitis **(b)**. Animals which were culture-positive or culture-negative formed a similar level of bone in both empty and CERAMENT groups. * p<0.05 using ANOVA + Student–Newman–Keuls post hoc method. Error bars represent ±1 standard deviation.

### 3.3 MicroCT (Figs. 5 and 6)

The bone volume fraction (BV / TV) of the region of interest in the CG group was greater than that of CBVF (0.21 vs. 0.17, p=0.021) and rats with empty defects (0.21 vs. 0.11, p<0.001). The BV / TV of CBVF-treated rats was greater than rats treated with empty defects (0.17 vs. 0.11, p<0.001) (Fig. 6a). In subgroup analysis, the CBVF group BV / TV of the ROI in rats without histological evidence of osteomyelitis was greater than that of rats with a substantial evidence of infection 0.19 vs. 0.14 (p=0.015) and similar to rats in the CG group, which all had no evidence of infection (0.19 vs. 0.21, p=0.32; Fig. 6b). Similarly, rats in the CBVF group without histological evidence of infection had a greater BV / TV than rats with empty defects (0.19 vs. 0.10, p<0.001), while rats with histological evidence of infection were similar to rats with an empty defect (0.14 vs. 0.10, p=0.14). A similar trend was not seen in culture-positive and culture-negative rats. There was no significant difference between culture-positive and culture-negative animals within CERAMENT and empty groups (Fig. 6c). Culture-positive CERAMENT samples had a greater BV / TV than culture-positive empty samples (0.19 vs. 0.09, p=0.009). Similarly culture-negative CERAMENT samples had a greater BV / TV than culture-negative empty samples (0.16 vs. 0.11, p=0.01).

## 4 Discussion

Osteomyelitis is a common infectious disease of bone which frequently necessitates debridement for appropriate management. The ensuing defect can require the use of a void filler. In this study we demonstrated that a calcium sulfate–hydroxyapatite cement with gentamicin (CERAMENT G, CG) decreased the rate of persistent infection and increased new bone growth as compared to both the same cement without gentamicin (CBVF) and an empty defect in a rat model of debrided osteomyelitis.

Two different methods of determining persistent infection were utilized in this study: bacterial culture and histological evidence of a osteomyelitis. By either criterion, no infection was detectable in CERAMENT G-treated (CG) rats at both 6 weeks and 6 months after implantation. By contrast, animals treated with an empty defect or a cement void filler without antibiotics (CBVF) had a 25 %–50 % rate of infection at 6 weeks after implantation. Previous studies examining the use of bone void fillers without antibiotics or defects left empty in osteomyelitis found high rates of persistent infection after debridement (Clawson et al., 1973; Gerhart et al., 1993; Jiang et al., 2012; Joosten et al., 2005; Kelly et al., 1970). By contrast, antibiotic-impregnated void fillers are known to be effective at treating osteomyelitis (Branstetter et al., 2009; McNally et al., 2016; Rand et al., 2015; Xie et al., 2009). While the majority of previous studies examined calcium sulfate carriers, this study utilized combination hydroxyapatite and calcium sulfate carrier, CERAMENT G. The advantages of these combination carriers include theoretically slower resorption, osteoconduction, controlled antibiotic release, and stronger mechanical properties (Iundusi et al., 2015; Nilsson et al., 2004, 2013; Raina et al., 2016; Solberg et al., 1999; Stravinskas et al., 2016). In contrast to our findings, a similar study carried out in pigs using CERAMENT G without the administration of systemic antibiotics found high rates of persistent infection with both limited and extensive debridement (Blirup-Plum et al., 2020). This study differed from ours as it utilized a foreign body and had a higher severity of osteomyelitis prior to debridement than that which was encountered in our study. Multiple clinical studies examining the use of CERAMENT G in clinical settings have demonstrated effective dead space management with low infection recurrence rate (Ferguson et al., 2019; McNally et al., 2016; Niazi et al., 2019).

In order to examine the osteoconductive properties of the groups, this study utilized microcomputed tomography to determine new bone formation within the debrided osteomyelitic cavity. New bone growth was higher in animals treated with CERAMENT G as compared with both the cement without antibiotics (CERAMENT, CBVF) and empty drill hole groups. Overall, defects that were implanted with cement formed more new bone in the defect compared with defects in the empty control group (BV / TV CG: 0.21 vs. CBVF: 0.17 vs. empty: 0.11). This result is consistent with the finding that resorbable cements enhance bone growth (Ferguson et al., 2019; Frankenburg et al., 1998; Hak, 2007). Interestingly, CERAMENT G improved bone growth even when compared with the CERAMENT (without antibiotic) group. While it is possible that this could be an unknown osteoinductive effect of gentamicin, it appears as if this is mediated primarily by the eradication of infection. The presence of persistent infection after debridement appeared to have a negative effect on bone growth in animals treated with cement without impregnated antibiotics. Subgroup analysis showed that animals without histological evidence of infection (indicated by a neutrophil-dominant inflammatory reaction ± necrotic bone) in the CBVF group had a bone volume fraction comparable to that of the CG-treated group (BV / TV of 0.19 vs. 0.21, respectively), while animals with infection had rates of new bone growth that were comparable to animals treated without any cement at all (BV / TV of 0.14 vs. 0.12, respectively). Thus, lack of histologically detectable infection was associated with increased new bone growth in the cement group without antibiotics and is likely the primary reason for improved bone growth in the CERAMENT G group as compared with the CBVF group. This trend was not seen among animals with an empty defect, and these rats showed similar levels of bone growth regardless of the presence or absence of histological evidence of infection. Animals subdivided by culture-positive and culture-negative status did not show the same trend, likely due to the decreased sensitivity of culture relative to histology for the detection of osteomyelitis (Lew and Waldvogel, 2004). No animals treated with CERAMENT G (CG) had any detectable infection so a similar comparison could not be drawn within this group. Both CERAMENT Bone Void Filler and CERAMENT G have been previously shown to facilitate new bone growth when utilized in uninfected environments, but literature which directly compared the two compounds in the absence of infection is not available (Nilsson et al., 2004; Oliver et al., 2020). To our knowledge there is no published literature detailing the relationship between persistent infection and decreased bone growth when bone graft substitutes are used in osteomyelitic environments.

We recognize that our study has several limitations. In clinical patients with osteomyelitis, surgical debridement is generally indicated in the presence of a significant amounts of necrotic bone. Abundant necrotic bone is typically produced in animal models by administering sclerosing agents or implanted foreign bodies. The downsides of using these agents are that they can be cumbersome or produce widespread osteomyelitis throughout the entire tibial medullary canal (Korkusuz et al., 1993; Rissing et al., 1985). We chose to utilize a model without the use of a sclerosing agent or a foreign body in order to induce a local mechanically stable, consistently sized osteomyelitis in the proximal tibia for ease of debridement and standardized evaluation of the region of interest (Fukushima et al., 2005). Even so, 68 % (49/72) of animals used in this study showed evidence of sequestrum, and 100 % had evidence of osteomyelitis on radiography immediately prior to debridement despite the lack of utilization of sclerosing agents. Having said this, at the end of our study, only 25 %–50 % of animals treated with an empty defect had histologically or microbiologically detectable infection, thus making assessing interventions more challenging given that the rats were likely to be able to clear the infection with debridement only.

Further, the animals used in this study did not receive any oral or intravenous antibiotics in contrast to the standard of care in real-world scenarios. While our model did not precisely mimic the clinical situation in this regard, we feel that the stark contrast in persistent infection between groups treated with and without local antibiotic therapy still represents an interesting and valid finding that displays bactericidal properties of the gentamicin within the ceramic composite. We utilized both culture and histology in order to detect infection, which both have separate disadvantages. It was expected that culture was a less sensitive marker for infection than histology especially because the method used for culturing bacteria in this study was non-destructive to the tissue and instead attempted to separate bacteria from the sample using mechanical agitation. This approach may have prevented the complete retrieval of bacteria from the bone tissue and thus lowered our sensitivity (Reizner et al., 2014). Conversely, a method relying on pulverization of the sample would have allowed a more thorough retrieval of bacteria for the purposes of culture but would have precluded histological or radiographic analysis. Thus we elected to utilize histology in addition to culture for a more robust detection of infection using multiple methods. These two methods did not differ significantly in the rate of positive results (p=0.2). Next, it is now known that *S. aureus* invades the osteocyte lacuno-canalicular network (OLCN) of live cortical bone during chronic osteomyelitis and that these bacteria are not susceptible to local high-dose antibiotic treatment. It is unclear whether our methods of detection would capture these bacteria if they were present in any of our groups (Masters et al., 2020; de Mesy Bentley et al., 2017, 2018; Schwarz et al., 2021). Lastly, in vivo gentamicin elution characteristics and systemic toxicity data were not recorded in these animals as they were outside the scope of the study and have been described elsewhere (Stravinskas et al., 2016).

## 5 Conclusion

The treatment of chronic osteomyelitis often leaves large defects that require bone grafting. Unfortunately, current bone graft materials have inadequacies including donor site morbidity, expense, or propensity to act as a nidus of infection. This study demonstrated decreased persistent infection rates and increased bone growth in debrided osteomyelitic cavities filled with a calcium sulfate–hydroxyapatite composite impregnated with gentamicin (CERAMENT G) when compared with an identical void filler without antibiotic impregnation and an empty defect. This study supports further investigation into the use of this material as a readily available void filler to be used in osteomyelitic environments after debridement.

## Data Availability

All data are available through Zenodo: (Dvorzhinskiy et al., 2021).
